# 

**Published:** 1865-03

**Authors:** 


### CHICAGO MEDICAL JOURNAL.

**Terms, $2.00 per Annum.**

### CONTENTS OF THE MARCH NUMBER.

PAGE.

ORIGINAL COMMUNICATIONS.

Official Investigation of the Charges Preferred against J. W. Freer, M. D., Surgeon of the Board of Enrollment, 1st District of Illinois, by the Homœopathists of Chicago ........ 97

Report on Orthopedic Surgery, made at the Annual Meeting of Illinois State Medical Society, convened in Chicago, May 3d, 1864. By David Prince, M. D., Jacksonville, Illinois........ 122

Milk Sickness. By. E. W. Boyles, of Clay City, Illinois........ 139

Communication. By Geo. D. Winch, Surgeon 42nd Ill. Vols., Cairo, Illinois........ 141

Illinois State Medical Society........ 142

